# Dual delivery gene-activated scaffold directs fibroblast activity and keratinocyte epithelization

**DOI:** 10.1063/5.0174122

**Published:** 2024-01-26

**Authors:** Ashang L. Laiva, Fergal J. O'Brien, Michael B. Keogh

**Affiliations:** 1 Tissue Engineering Research Group-Bahrain, Royal College of Surgeons in Ireland, Adliya, Kingdom of Bahrain; 2 Tissue Engineering Research Group, Department of Anatomy and Regenerative Medicine, Royal College of Surgeons in Ireland, 123 St. Stephen's Green, Dublin 2, Ireland; 3 Trinity Centre for Biomedical Engineering, Trinity Biomedical Sciences Institute, Trinity College Dublin, Dublin 2, Ireland; 4 Advanced Materials and Bioengineering Research Centre, Royal College of Surgeons in Ireland and Trinity College Dublin, Dublin, Ireland

## Abstract

Fibroblasts are the most abundant cell type in dermal skin and keratinocytes are the most abundant cell type in the epidermis; both play a crucial role in wound remodeling and maturation. We aim to assess the functionality of a novel dual gene activated scaffold (GAS) on human adult dermal fibroblasts (hDFs) and see how the secretome produced could affect human dermal microvascular endothelial cells (HDMVECs) and human epidermal keratinocyte (hEKs) growth and epithelization. Our GAS is a collagen chondroitin sulfate scaffold loaded with pro-angiogenic stromal derived factor (SDF-1α) and/or an anti-aging β-Klotho plasmids. hDFs were grown on GAS for two weeks and compared to gene-free scaffolds. GAS produced a significantly better healing outcome in the fibroblasts than in the gene-free scaffold group. Among the GAS groups, the dual GAS induced the most potent pro-regenerative maturation in fibroblasts with a downregulation in proliferation (twofold, p < 0.05), fibrotic remodeling regulators TGF-β1 (1.43-fold, p < 0.01) and CTGF (1.4-fold, p < 0.05), fibrotic cellular protein α-SMA (twofold, p < 0.05), and fibronectin matrix deposition (twofold, p < 0.05). The dual GAS secretome also showed enhancements of paracrine keratinocyte pro-epithelializing ability (1.3-fold, p < 0.05); basement membrane regeneration through laminin (6.4-fold, p < 0.005) and collagen IV (8.7-fold, p < 0.005) deposition. Our findings demonstrate enhanced responses in dual GAS containing hDFs by proangiogenic SDF-1α and β-Klotho anti-fibrotic rejuvenating activities. This was demonstrated by activating hDFs on dual GAS to become anti-fibrotic in nature while eliciting wound repair basement membrane proteins; enhancing a proangiogenic HDMVECs paracrine signaling and greater epithelisation of hEKs.

## I. INTRODUCTION

Skin is the largest organ of the body that acts as a barrier to protect internal organs against external harmful stimuli. Upon injury, cells in the wound initiate a series of healing events to restore the skin's normal structure and functioning.^1^ However, the functional integrity of the skin declines with age and other metabolic-associated diseases such as diabetes, reducing skin's healing ability.^2^ Moreover, in adults, skin cells including dermal fibroblasts may undertake a fibrotic pathway to accelerate wound contraction and matrix deposition.^3^ Clinically, this can result in a functionally inferior scar tissue formation which may lead to pain and restricted movement, particularly when located near the joints.^4^ To date, controlling the scarring process in skin tissue remains a major medical challenge.

Wounds that are too large to heal on their own generally require allogenic skin grafts that are accessed from unaffected healthy regions of a matched donor.^5^ However, in cases when an allogenic graft transplantation is not plausible, surgeons may utilize the application of synthetic biomaterial-based scaffolds. As an example, Integra's dermal regeneration template (DRT) is one of the first and a popular acellular skin mimetic scaffold used for the treatment of large skin wounds.^6^ Integra's DRT is rich in type 1 collagen and chondroitin sulfate, which are the major extracellular matrix (ECM) components of the skin; when transplanted into the wounds, skin cells are attracted to grow into the scaffold and promote repair.^7^ Another category of dermal regenerative product is the bioengineered skin equivalent graft.^8^ Company such as Organogenesis, Inc., has been developing bioengineered dermal grafts using a combination of neonatal fibroblasts and type 1 collagen scaffolds, for example, Apligraf^®^.^9^ One advantage of the bioengineering technique is that it allows the creation of a more complex scaffold through the deposition of cell-derived minor ECM components such as collagen IV and laminin.^10^ Collagen IV and laminin form major components of the BM, which are essential for epidermal regeneration.^11^ Moreover, dermal fibroblasts secrete paracrine factors that can favorably modulate the wound environment to promote wound repair.^8^ A controlled balance of these chemokines is required to prevent scar tissue formation while allowing for keratinocyte epithelization.

While developing bioengineered constructs, cells can be manipulated to produce a more robust healing response. A common approach is stimulating the cells in a growth factor rich environment; however, this is not ideal due to cost, dosage rates, and half-life related issues.^12^ Alternative strategies, which have shown promise, involve the generation of cell instructive scaffolds using gene therapy.^13^ Gene therapy uses therapeutic plasmids of DNA or RNA encoding the gene of interest coupled with a vector to improve efficient transgene expression of chemokines. The nanoparticles (polymers or peptides) plasmid complexes often referred to as the gene activated scaffold (GAS).^14^

Our group's research focuses on the use of non-viral vector-based GAS for tissue repair. We have found for example that the non-viral vector polyethyleneimine (PEI) is an efficient gene-delivery vector for transfecting a broad spectrum of cells.^15–18^ Consequently, PEI-based nanoparticles are often used as a vector within GAS.

It is well established that there are several factors involved during the wound healing process to maintain a controlled tissue formation via neo-angiogenesis and reduced contracture and fibrosis. In skin wounds, stromal-derived factor-1 alpha (SDF-1α) expression elevates at the wound margin and is secreted to recruit endothelial progenitor cells that promotes angiogenesis.^19^ Previously, we have also shown that SDF-1α incorporated into a GAS could significantly enhance pro-angiogenic cellular responses in a multitude of cells.^16–18,20^

Another important factor for wound healing is controlled fibrosis. Using the anti-aging and pro-rejuvenating molecule β-klotho, we have shown β-Klotho GAS could significantly enhance human diabetic adipose-derived stem cells (ADSCs) stemness by activating wound healing pathways similar to healthy cells on gene free scaffolds.^21^ We, therefore, hypothize the combination of a pro-angiogenic SDF plasmid and an anti-fibrotic stem cell activating β-klotho plasmid on our collagen chondroitin scaffold may give optimal wound healing abilities for skin regeneration. To examine this, we investigate the effects of a dual GAS on dermal human adult fibroblasts the main dermal cell type and study what paracrine bioactivity from our GAS may have on mature skin keratocytes the main epidermal cell type.

## II. RESULTS

### A. The dual GAS reduces adult hDF proliferation and downregulates fibrotic matrix remodeling factors toward maturation

**FIG. 1.**
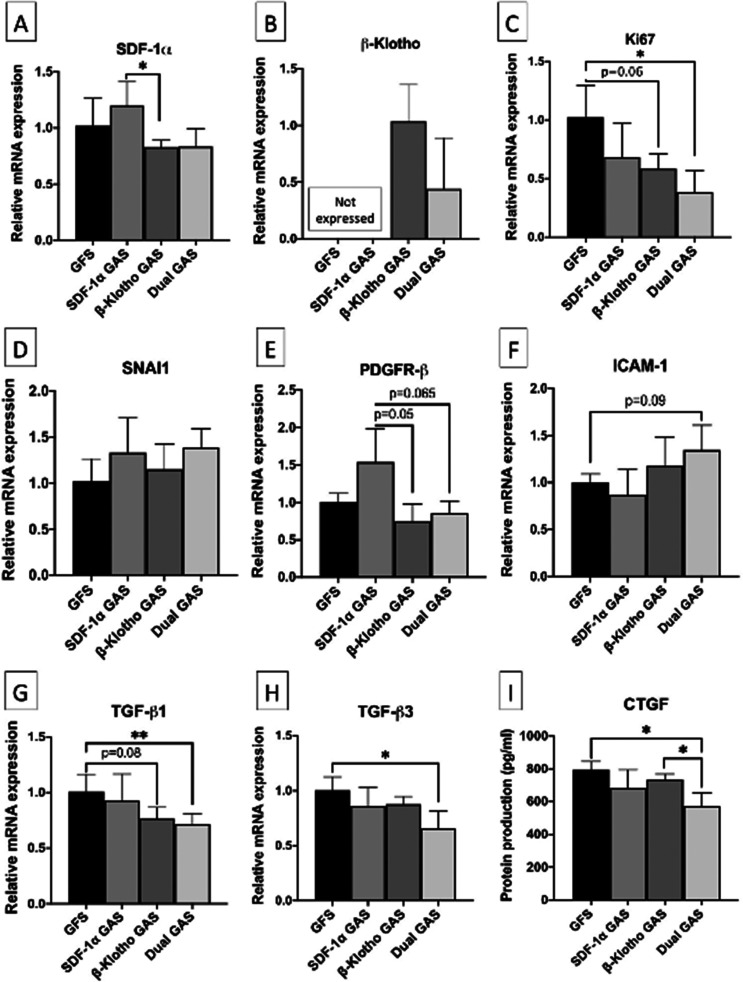
Gene expression analysis of reparative maturation factors and CTGF protein production in hDFs at day 14. (a) and (b) show the expressions of transgenes SDF-1α and β-Klotho in the GAS vs gene-free scaffolds. (c) show the reduced hDFs proliferation in the GAS than gene-free scaffold. (d) and (e) show the expression of mesenchymal functional regulators. hDFs in SDF-1α GAS showed the highest pro-fibrotic PDGFR-β expression. (f) hDFs in the dual GAS demonstrated the highest induction of pro-matrix remodeling ICAM-1 gene. (g)–(i) show the expression of TGF-βs and its downstream effector CTGF. hDFs in the dual GAS expressed the least of the TGF-βs and CTGF. ^*^ and ^**^ indicates statistical significance of p < 0.05 and p < 0.01, respectively (n = 3). The acronyms GFS and GAS stand for gene-free scaffold and GAS, respectively.

Following day 14 culture all GAS remained viable with favorable number of cells on GAS remaining viable (≥72%) relative to gene-free scaffolds. Gene expression analysis first revealed that the human adult dermal fibroblasts (hDFs) intrinsically express SDF-1α but not β-Klotho. Only the GAS functionalized with the β-Klotho gene promoted β-Klotho expression in the hDFs. Also, while the β-Klotho or dual GAS supported basal SDF-1α expression in the hDFs, SDF-1α GAS did not promote β-Klotho expression in the hDFs. Among the GAS, only the dual GAS induced a robust reparative maturation response in the hDFs, compared to the gene-free scaffold [Figs. 1(a)–1(c)]. The main outcomes in the dual GAS group include significant reductions in cell proliferation (Ki-67; p < 0.05), pro-fibrogenic (TGF-β1; p < 0.01), as well as anti-fibrogenic (TGF-β3; p < 0.05) factors. In determining pro-fibrotic CTGF protein production by the hDFs (Fig. 1), we also noted that the dual GAS group secreted the least of the CTGF protein (572 ± 82 pg/ml; p < 0.05 vs gene-free scaffold group). This response was further accompanied by a modest 35% (p < 0.09) increase in pro-matrix remodeling factor ICAM-1 in the hDFs without changes in the basal mesenchymal gene expressions, including the pro-fibrotic PDGFR-β [Figs. 1(d) and 1(e)].

### B. Dual GAS promotes anti-fibrogenic differentiation of adult hDFs

**FIG. 2.**
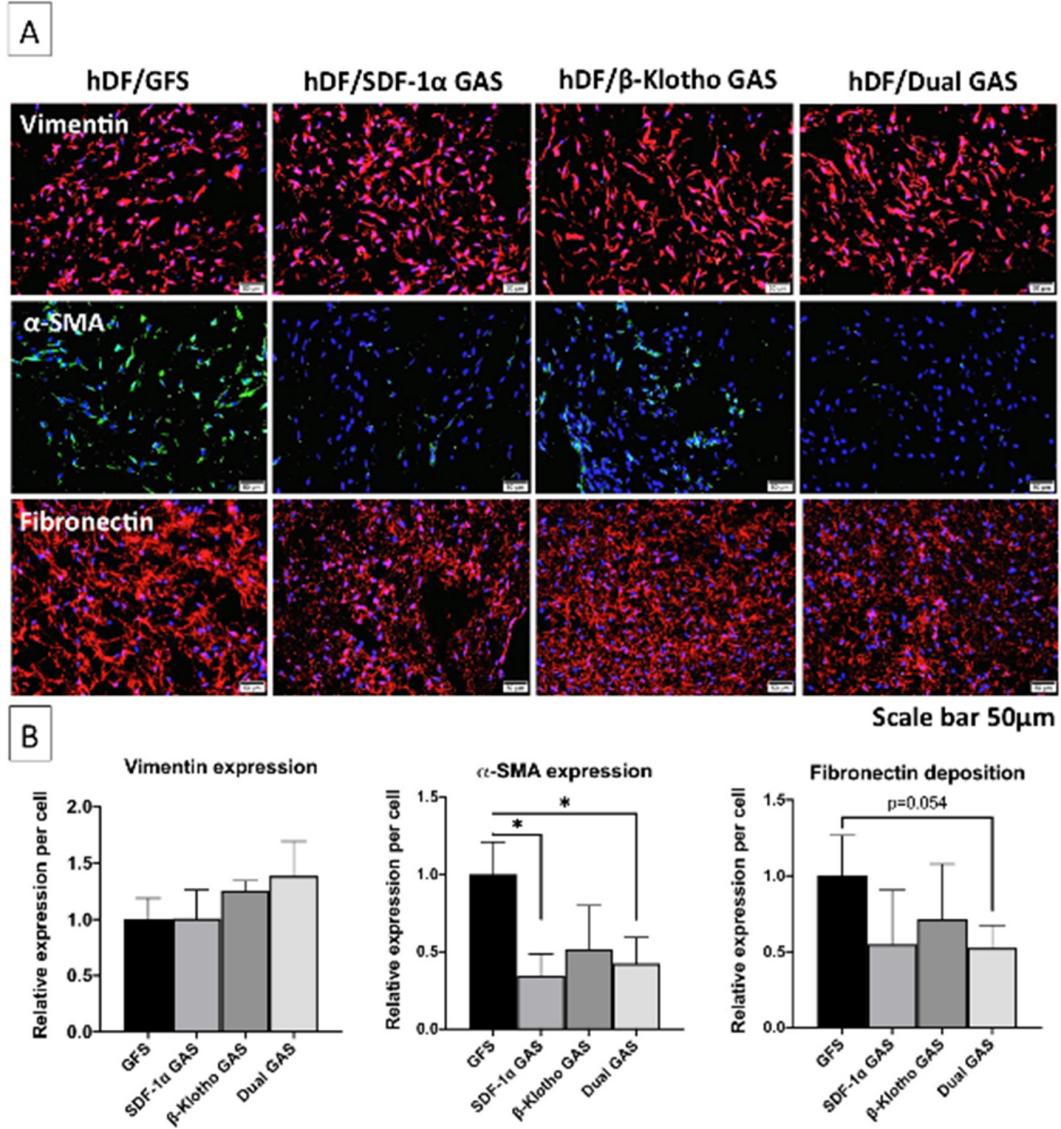
Immunofluorescence analysis of mesenchymal fibroblasts markers involved in fibrotic maturation. (a) Immunofluorescent images of mesenchymal markers expression in the hDFs at day 14. hDFs in all the groups showed similar levels of mesenchymal marker vimentin. hDFs in the gene-free scaffold showed the highest expression of pro-fibrotic markers α-SMA and fibronectin. (b) Semi-quantitative analysis revealed that the hDFs in all the GAS expressed 50% lower α-SMA and fibronectin than in the gene-free scaffold. * indicates statistical significance of p < 0.05 (n = 3). The acronyms GFS and GAS stand for gene-free scaffold and GAS, respectively.

Having determined that the hDFs in the dual GAS produce an anti-fibrogenic maturation response at the transcriptional level, we then studied key fibroblast markers involved in fibrotic maturation using immunofluorescence (Fig. 2). Toward maturation, the hDFs in the gene-free scaffold showed abundant α-SMA and fibronectin expression. While α-SMA expression was predominantly cellular, fibronectin was abundantly detected as an extracellular fibrous matrix [Fig. 2(a)]. However, we noted a significant reduction in the fibrogenic differentiation of the hDFs in the dual GAS. Semi-quantitative analysis [Fig. 2(b)] revealed that the expression of fibrogenic markers α-SMA (p < 0.05) and fibronectin (p < 0.054) was lower by 50% than in the gene-free scaffold group. This antifibrotic effect was supported by the noted reduction in GAS size by 21% gene-free and by ≤5% for SDF-1α, β-Klotho and Dual GAS groups.

Meanwhile, in association with the modest anti-fibrogenic transcriptional response, we also noted a robust anti-fibrogenic response in the SDF-1α and β-Klotho GAS groups. The hDFs in the SDF-1α and β-Klotho GAS barely expressed α-SMA and minimally deposited the fibronectin matrix composed of short, fragmented networks. It is also worth noting that the hDFs in all the groups retained their quiescent mesenchymal state, as shown by their basal vimentin expression levels [Fig. 2(b)].

### C. Dual GAS enhances human epidermal keratinocytes epithelialization while supporting human dermal microvascular endothelial angiogenesis

**FIG. 3.**
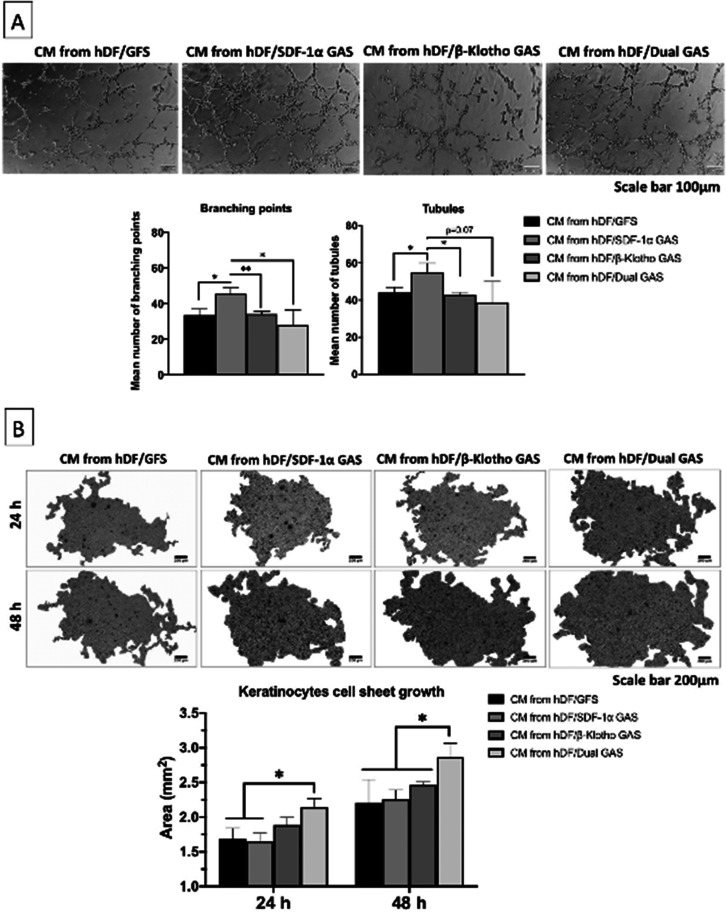
Pro-angiogenic and pro-epithelializing bioactivity analyses of hDF secretome. (a) Pro-angiogenic impact of hDFs' CM on human dermal microvascular endothelial cells. CM derived from the SDF-1α GAS group induced the strongest pro-angiogenic response in the endothelial cells, evidenced by increased number of endothelial tubules and branching. (b) Pro-epithelializing impact of hDFs' CM on human epidermal keratinocytes. hDFs' CM promoted keratinocytes epithelialization temporally. Relatively, culture media derived from the dual GAS group induced the most potent pro-epithelializing response in the keratinocytes. ^*^ and ^**^ indicates statistical significance of p < 0.05 and p < 0.01, respectively (n = 3). The acronyms GFS and GAS stand for gene-free scaffold and GAS, respectively.

Having confirmed the anti-fibrogenic differentiation of the hDFs in the GAS, we next assessed the bioactivity of the secretome produced by the hDFs toward dermal microvascular endothelial angiogenesis and epidermal keratinocytes epithelialization. First, we evaluated the microvascular endothelial angiogenesis in response to the hDFs' CM [Fig. 3(a)]. All the groups demonstrated the ability to support endothelial angiogenesis. Tubulogenesis of the endothelial cells occurred within 6 h of CM stimulation, followed by branching of tubular networks. However, among the groups, the secretome produced by the SDF-1α GAS group exerted the most potent pro-angiogenic response characterized by enhanced endothelial tubulogenesis (55 ± 4) and branching (46 ± 3). Angiogenic response induced by the SDF-1α GAS group was significantly (p < 0.05) higher than all the groups. The gene-free scaffold group and the other GAS groups showed no differences in the secretome pro-angiogenic bioactivity.

The second bioactivity test was to assess the secretome's pro-epithelializing potency. Upon CM treatment, the epidermal keratinocytes self-assembled into cell sheets and continued to expand until 48 h post-treatment [Fig. 3(b)]. However, contrary to the angiogenic findings, the dual GAS group exerted the most potent pro-epithelializing effect in the keratinocytes. The keratinocyte cell sheet grew significantly (p < 0.05) larger at both time points than gene-free and SDF-1α GAS groups. Cell sheet area in the dual GAS group stimulated keratinocytes grew from 2.1 ± 0.13 mm^2^ at 24 h to 2.9 ± 0.2 mm^2^ by 48 h. Keratinocytes stimulated by the gene-free scaffold group formed a much smaller cell sheet of 1.65 ± 0.12 mm^2^ at 24 h that grew to 2.25 ± 0.14 mm^2^ by 48 h.

### D. Dual GAS enhances basement membrane production toward tissue reconstruction in hDFs

**FIG. 4.**
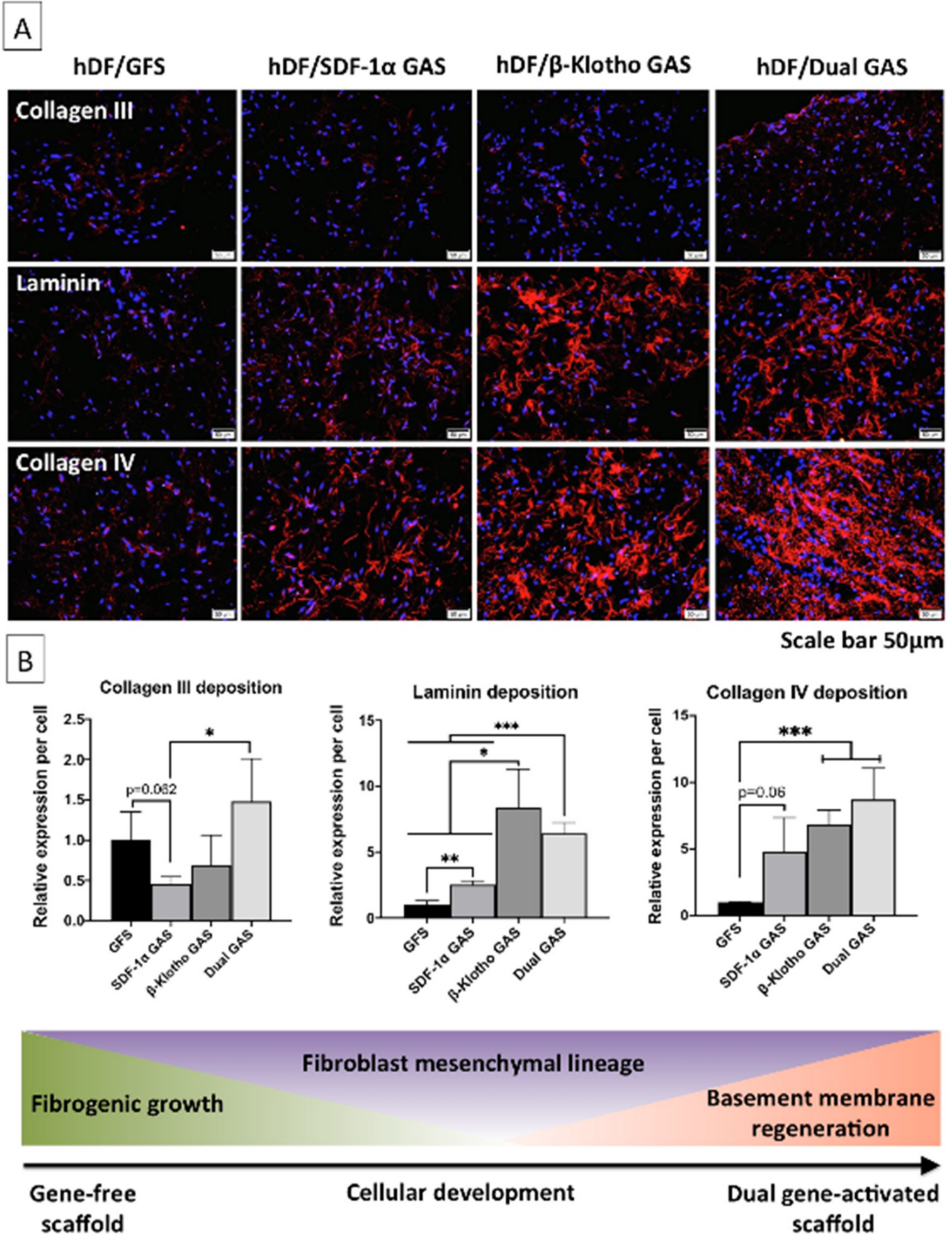
Immunofluorescence analysis of reparative matrix deposition toward maturation. (a) Immunofluorescent images of early granulation tissue protein collagen III and BM proteins laminin and collagen IV deposition. hDFs in all the GAS abundantly deposited the BM proteins laminin and collagen IV. (b) Semi-quantitative analysis revealed that hDFs in the β-Klotho and dual GAS regenerated the highest amounts of laminin and collagen IV matrix. SDF-1α GAS group showed the weakest deposition of collagen III matrix. ^*^, ^**^, ^***^ indicates statistical significance of p < 0.05, p < 0.01, and p < 0.005, respectively (n = 3). The acronyms GFS and GAS stand for gene-free scaffold and GAS, respectively.

After determining the anti-fibrogenic pro-epithelializing ability of the GAS, we sought to assess pro-regenerative hDF maturation by imaging granulation tissue matrix deposition (Fig. 4). Overall, the gene-free scaffold group deposited the least of the granulation tissue matrix proteins [Fig. 4(a)]. In general, collagen III deposition was weak in all the groups; however, the lowest deposition occurred in the SDF-1α GAS group. Collagen III deposition in the SDF-1α GAS group was lower by about 54% and 70% (p < 0.05) than in the gene-free and dual GAS groups, respectively. Nevertheless, SDF-1α GAS group richly enhanced the production of BM proteins laminin (2.52-fold; p < 0.01) and collagen IV (4.8-fold; p < 0.06) than the gene-free scaffold group [Fig. 4(b)].

The BM regeneration was more pronounced in the β-Klotho and dual GAS groups. Relative to the gene-free scaffold group, β-Klotho GAS stimulated laminin and collagen IV deposition in the hDFs by 8.4-fold and 6.8-fold, respectively. Meanwhile, the dual GAS group deposited 6.4 and 8.7-folds of laminin and collagen IV, respectively. Also, the β-Klotho and dual GAS groups deposited significantly (p < 0.05) higher amounts of laminin than the SDF-1α GAS group. A significantly greater reconstruction of the granulation tissue was observed in β-Klotho and dual GAS groups through enhanced BM regeneration.

## III. DISCUSSION

Controlled wound healing is crucial to restoring the skin's normal structure.^22^ Under certain circumstances for example post debridement of a chronic ulcer tissue repair can undertake a fibrotic pathway in order to accelerate faster wound closure via irregular wound contraction and fibrous scar tissue formation.^3^ These fibrotic responses may limit the tissues ability to heal in a controlled manner and can significantly affect patients' quality of life.^23^ Dermal fibroblasts are the major contributors to this scarring process and keratinocytes are the major cell type involved in epidermal epithelization.^3^ Therefore, this study investigated the potency of a dual GAS containing pro-angiogenic and pro-rejuvenate properties to promote healthy healing response in human adult dermal fibroblasts and how their secretome may influence human keratinocyte activities.

We demonstrated how fibroblasts adopted a more controlled anti-fibrogenic pathway toward maturation, evidenced by retardation of cell proliferation and downregulation of fibrotic remodeling factors, stimulation of keratinocytes epithelialization, and enhanced regeneration of BM. The matured fibroblasts maintained their mesenchymal traits and also supported human dermal microvascular endothelial angiogenesis. These fibroblast responses collectively indicate that the dual GAS is a potent biomaterial scaffold for healthy dermal regeneration; this may be beneficial clinically in treating persistent non healing ulcers.

Dermal fibroblasts are known to express high basal levels of SDF-1α mRNA;^24^ therefore, transfection may not sustain SDF-1α overexpression at maturation. Whereas, β-Klotho is expressed at relatively low levels in the skin but may become elevated following wounding to facilitate a FGF21 response.^25,26^ We also showed that the hDFs could efficiently transcribe the β-Klotho gene when grown on the β-Klotho and dual GAS. It is also worth noting that SDF-1α overexpression did not stimulate β-Klotho transcription in the hDFs, while β-Klotho overexpression stably supported SDF-1α expression. This finding suggested that the co-expression of β-Klotho could raise the functional potency of SDF-1α overexpressing hDFs.

TGF-β1 is a potent inducer of myofibroblasts differentiation in the fibroblasts, phenotypically characterized by the α-SMA expression.^27,28^ The CTGF, a downstream mediator of TGF-β1, is an endogenous regulator of fibronectin expression.^29^ These regulators are essential for the onset of the fibroproliferative switch of the fibroblasts during healing; however, long-term activation of the fibroproliferative state can be detrimental and lead to fibrosis.^27^ Our finding indicates GAS could activate the hDFs in a healthier anti-fibrotic state than the gene-free scaffold control. GAS demonstrated a 50% reduction in the pro-fibrotic α-SMA and fibronectin expression. Specifically, hDFs grown on the dual GAS demonstrated over 50% reduction in cell proliferation, which has been shown to be crucial for fibroblast quiescent state transition to engage in secretome driven regeneration and restore tissue homeostasis.^30^

In wound healing, SDF-1α is known more commonly as an angiogenesis promoter but Rabbany *et al.* has shown how SDF-1α plasmid delivery using alginate hydrogel can suppress α-SMA and promoted scarless dermal healing.^31^ Sundararaman *et al.* also showed that SDF-1α plasmid delivery could suppress cardiac fibrosis post-myocardial infarction in rat hearts.^32^ Although little is known about the anti-fibrotic role of SDF-1α, SDF-1α may inhibit TGF-β1 signaling through activation of its receptor CXCR7,^33^ which can suppress the pro-fibrotic Wnt/β-catenin pathway.^34^ Suppressing the Wnt/β-catenin signaling is also one of the prominent mechanisms of Klotho-driven anti-fibrotic response.^35^ As both SDF-1α and Klotho target TGF-β1, the enhanced transcriptional suppression of TGF-β1 in the dual GAS group indicates the synergistic functioning of the two transgenes in the hDFs. Similar to TGF-β1, we also noted a coordinated downregulation of TGF-β3 mRNA in the dual GAS group. The coordinated regulation of TGF-βs 1 and 3 is essential to drive normal healing in human adult skin wounds.^36^ However, contrary to popular belief about TGF-β3's anti-fibrotic role, recent evidence indicates that high levels of TGF-β3 mRNA correlates with increased scarring in human adult skin wounds.^36^ Moreover, Quan *et al.* also found that silencing the TGF-β1 or 3 genes can significantly reduce the pro-fibrotic CTGF production by 30% in human adult dermal fibroblasts and that the response amplifies by twofold (60%) when both the TGF-βs are suppressed simultaneously.^37^ Taken together, our findings of the matrix remodeling regulators strongly imply a tight regulation of the anti-fibrotic TGF-β pathway in the dual GAS group.

While angiogenesis is crucial for the onset of healing, its resolution is equally vital in wound maturation to limit scarring.^38^ One mechanism through which abundant angiogenesis could increase scarring is by recruiting pericytes that can adopt a myofibroblast phenotype.^39^ Our finding shows that the angiogenesis is well moderated in the β-Klotho and dual GAS groups toward maturation. In these groups, the dermal microvascular endothelial angiogenesis involved tubulogenesis but lacked robust endothelial network connectivity similar to the gene-free scaffold group. However, SDF-1α GAS group induced an extended angiogenic activity in the dermal microvascular endothelial cells. We do not yet understand the crosstalk between pro-angiogenic dermal endothelial cells and pericytes; however, we previously found that the co-culture of human umbilical vein endothelial cells with ADSCs (pericytes) on the SDF-1α GAS undergoes enhanced pro-vasculogenic maturation through enhanced eNOS expression.^18^ The eNOS is also a crucial anti-fibrotic effector in wound healing.^40,41^ Hence, the extended angiogenic bioactivity in the SDF-1α GAS group may not drive a higher scarring response at maturation.

Another healing event that accompanies angiogenesis is epithelialization. Rapid epithelialization is an important predictor of scarless healing.^42^ For instance, scarless healing in oral mucosa typically lacks angiogenesis^43^ and is dominantly driven by epithelialization.^44^ Fibroblasts-derived soluble factors are the main effectors of keratinocytes re-epithelialization.^45^ Our finding also shows that the hDFs have a strong pro-epithelializing impact on keratinocytes. The collected GAS secretome promoted rapid keratinocytes cell sheet assembly. Keratinocytes stimulated by the dual GAS group showed the most rapid growth of cell sheets, covering at least 2.1 mm^2^ in the area at 24 h that grew to ∼3 mm^2^ in 48 h.

In addition to re-epithelialization, proper BM regeneration is essential for epidermal homeostasis maintenance.^46^ Abnormalities in the BM can significantly delay epithelialization and promote scarring.^47^ Moreover, the BM is highly susceptible to damage by sun exposure, and its regenerative capacity significantly decreases with aging.^48,49^ Restoration of BM is thus a critical target in wound healing strategies. Fibroblasts seeded on collagen chondroitin sulfate scaffolds have already been proposed as a pro-BM regenerative construct.^50^ Studies have also shown that fibroblasts-seeded collagen scaffolds could restore normal-like epidermal rete ridge-like structure post-healing,^51^ including epidermal appendages regeneration such as hair follicles.^52^ Here, we also show that the GAS can significantly enhance the deposition of primary BM components laminin and collagen IV in the hDFs. Relative to the SDF-1α GAS, the β-Klotho and dual GAS demonstrated higher BM regenerative potency. The enhanced BM regenerative potency of the β-Klotho GAS group is also in line with our findings from the previous study that used human ADSCs.^53^ As the BM regeneration is higher in the β-Klotho GAS group than in the SDF-1α GAS group, it is justifiable that the enhanced response in the dual GAS group is mediated by β-Klotho overexpression in the hDFs. The increased deposition of anti-scarring early granulation protein collagen III in the dual than in the SDF-1α GAS group further supports that the β-Klotho overexpression raises the functional potency of SDF-1α expressing hDFs.

## IV. CONCLUSION

In this study, we showed that the dual angiogenic SDF-1α and anti-fibrotic β-Klotho genes-loaded collagen chondroitin sulfate scaffold could tremendously improve the reparative maturation of human dermal fibroblasts (hDFs) and activate both human adult dermal microvascular endothelial cells (HDMVECs) and human epidermal keratinocytes (hEKs). We specifically showed that the human dermal fibroblasts in the dual GAS can transition to a quiescent state by downregulating pro-fibrotic factors expression and fibrogenic differentiation and more efficiently regeneration of the basement membrane. The secretome from the hDF loaded dual GAS promoted wound healing by enhanced proangiogenic branching of HDMVECs and greater keratinocyte epithelialization; potentially directing toward scarless wound healing and regeneration.

## V. METHODS

### A. Preparation of GAS

The GAS was developed using a well-established process as described by O' Brien *et al.*^13^ Briefly, a blended slurry of bovine tendon type 1 collagen and shark cartilage chondroitin-6-sulfate (Sigma, UK) was freeze dried using an optimized freeze-drying process to produce solid highly porous scaffolds (99.9% porosity) and a mechanical stiffness of 0.4 kPA. Scaffolds were cut to shape using a 10 mm surgical punch. The freeze-dried scaffolds were then treated at 105 °C under vacuum for sterilization and scaffold cross-linking. After the heat treatment, the scaffolds were further chemically cross-linked with 14 mM N-(3-Dimethylaminopropyl)-N′-ethylcarbodiimide hydrochloride and 5.5 mM N-Hydroxysuccinimide (EDC/NHS) (Sigma, UK) solution to enhance their mechanical stability. The cross-linked scaffolds were then washed with PBS (Gibco, UK) to remove residual chemicals. Gene-activation of the cross-linked scaffolds were then carried out by soak-loading polyethyleneimine (PEI) polyplex nanoparticles (100–150 nm in diameter) on to the scaffolds for 40 min.^14^ The polyplexes are formulated through self-assembling a pre-determined volume of branched 25 kDa solution with plasmid DNA (pDNA) encoding the therapeutic gene to give a final nitrogen to phosphate (N/P) ratio of 10. We use a total of 2 *μ*g pDNA per scaffold to develop the GAS. For dual GAS, 1 *μ*g pDNA of each of the therapeutic genes are mixed with the PEI solution to form the polyplexes and soak-loaded on to the scaffolds. The nanoparticles soak-loading is performed by pipetting equal volumes of the polyplex solution per side of the scaffold. In this study, we prepared four scaffold groups—(1) gene-free scaffold (as prepared cross-linked scaffold), (2) SDF-1α GAS, (3) β-Klotho GAS, and (4) SDF-1α/β-Klotho GAS (dual GAS). The SDF-1α and β-Klotho plasmids were obtained from InvioGen and SinoBiological, Beijing, China, respectively. The SDF-1α plasmid contains the human elongation factor-1α (hEF1) as the core promoter while the β-Klotho plasmid contains the cytomegalovirus (CMV3) promoter to induce high-level stable and transient expression of the encoded gene in mammalian cells.

### B. Cell seeding on GAS

Human adult dermal fibroblasts (hDFs) derived from a 39-year old male donor (Cat No. 10HU-014) was purchased from iXCells Biotechnologies, USA. The hDFs were expanded to passage 4 in the fibroblasts growth medium (Cat no. MD0011) supplied by the company. For experiments, a total of 5 × 10^5^ hDF (2.5 × 10^5^ per side) were seeded per gene-free or GAS. After letting the cells settle for about 20 min, 2 ml of transfection media OptiMEM (Gibco, UK) was added, and the cellularized scaffolds were incubated at 37 °C for 24 h. After the 24 h incubation, the cellularized gene-free or GAS were transferred into new 12-well plates and fed with 2 ml of hDF growth medium. Media change was then performed every 3–4 days until day 14 by collecting 1 ml of the conditioned media (CM) and replacing it with equal volume of fresh media. Diameters of all GAS 10 mm disks were measured on day 14. All CM were stored at −80 °C until analysis.

Cell viability following day 14 was assessed using the colorimetric MTS assay (CellTiter 96^®^ AQ_ueous_ One Solution, Promega, Madison, WI, USA). Briefly, at 1, 3, and 7 days post-transfection, 20 *μ*l of the MTS reagent was added to the cells in 100 *μ*l of media, and incubated for 4 h at 37 °C. Intensity of the resulting color was measured at an absorbance of 490 nm using a Varioskan Flash multimode plate reader (Fisher Scientific, Ireland). Cell viability percentage (%) was determined according to the equation (absorbance_[transfected]_/absorbance_[control]_) × 100, keeping the untransfected cells as 100% viability control.

### C. qRT-PCR analyses to determine transcriptional regulation in fibroblasts for wound healing

Cells were harvested at day 14 post-seeding on the gene-free or GAS. The cells were first lysed using the Qiazol lysis reagent (Qiagen, UK). Chloroform was then added to separate the cell lysate into protein, DNA and RNA phases. Using the RNeasy Kit (Qiagen, UK), the RNA was extracted and their quality and quantity were determined using a Multiskan Go plate reader (Thermo Scientific, UK) with the absorbance set at 260 nm. Genomic DNA was then removed by mixing the RNA with a genomic DNA wipeout buffer (Qiagen, UK) and heating to 42 °C for 2 min. Subsequently, reverse transcription was performed to prepare the cDNA. Duplicates of cDNA per replicate (n = 3) were loaded into the qRT-PCR plates and then the assay was run using the primers listed in Table I. The fold change in mRNA expression relative to the cells on gene-free scaffold was calculated using the 2^−ΔΔ^CT method from averages of three replicates per group. Human GAPDH (Hs_GAPDH_1_SG, Cat. No. QT00079247) was used as the housekeeping gene. All the primers were obtained from Qiagen, UK.

**TABLE I.** List of functional genes associated with hDFs' maturation.

Indicators	Primer (Catalog no.)	Encoded gene	Functional roles
Transgene activation and proliferation	Hs_CXCL12_1_SG (QT00087591)	Stromal-derived factor-1 alpha (SDF-1α)	Promotes angiogenesis and cellular homeostasis^54^
	Hs_KLB_4_SG (QT02454977)	Beta Klotho (β-Klotho)	Cellular rejuvenation and matrix regeneration [T]
	Hs_MKI67_1_SG (QT00014203)	Marker of proliferation (Ki-67)	Indicator of pro-liferation-quiescence transition^25^
Mesenchymal markers	Hs_SNAI1_1_SG (QT00010010)	Snail family transcriptional repressor 1 (SNAI1)	Maintenance of mesenchymal cell function and matrix ingrowth^26^
	Hs_PDGFRB_1_SG (QT00082327)	Platelet-derived growth factor receptor beta (PDGFR-β)	Promotes fibroblasts migration and fibrosis^30,53^
Matrix remodeling regulators	Hs_ICAM1 _1_SG (QT00074900)	Intercellular adhesion molecule 1 (ICAM-1)	Pro-epithelializing matrix remodeling factor^55,56^
	Hs_TGFB1_1_SG (QT00000728)	Transforming growth factor beta 1 (TGF-β1)	Pro-fibrotic matrix remodeling factor^57^
	Hs_TGFB3_1_SG (QT00001302)	Transforming growth factor beta 3 (TGF-β3)	Anti-fibrotic matrix remodeling factor^58,59^

### D. ELISA for quantitative determination of connective tissue growth factor (CTGF) production in hDF constructs

Next, we sought to determine cellular maturation response by assessing the production of CTGF, the global mediator of tissue remodeling and fibrosis in wound healing.^29,60^ To perform the ELISA, we loaded 100 *μ*l of the CM collected on the 14th day of culture (n = 3/group) into duplicate wells of CTGF ELISA (DY9190-05, R&D Systems, UK) plate. Assays were then run according to the manufacturer's instructions. A Multiskan Go plate reader (Thermo Scientific, UK) was used to measure the absorbance of the samples at 450 nm. The quantity of CTGF present was deduced by calculating against a standard curve.

### E. Paracrine bioactivity of GAS analyses

#### 1. Dermal microvascular endothelial pro-angiogenic bioactivity

Next, we studied the angiogenic bioactivity of the secretome produced by the hDF laden gene-free and GAS. The Matrigel™ assay was used to determine angiogenic growth of human adult dermal microvascular endothelial cells (HDMVECs) derived from a 25-year old male donor. The HDMVECs were first expanded to passage 4 using the endothelial growth media. For the Matrigel assay, wells of a 48-well plate were coated with 120 *μ*l of Matrigel per well and incubated for 30 min at 37 °C to allow gelation of the Matrigel. After gelation, 300 *μ*l of CM from each of the groups was loaded into the wells. 2 × 10^4^ HDMVECs were then seeded on to each well (n = 3/group). At 6 h post-exposure to CM, images of the morphological changes in the endothelial cells were captured using 10× objective of an inverted microscope (IX73, Olympus, Japan) and the mean number of tubules and branching points were counted using the ImageJ software (ImageJ, NIH, Maryland, USA). The HDMVECs (Cat No. 10HU-019) and endothelial growth media (Cat No. MD-0010) was supplied by iXcells Biotechnlogies, USA.

#### 2. Epidermal keratinocytes pro-epithelializing bioactivity

After the angiogenesis assay, we investigated the pro-epithelializing bioactivity of the hDFs's secretome on human adult epidermal keratinocytes (hEKs) derived from a 34 year old female donor (iXcells Biotechnologues, Cat no. 10HU-225). For this assay, 200 *μ*l CM from each of the hdF laden scaffolds were first loaded into each well of a 48-well plate. Next, 1.2 × 10^4^ of third passage hEKs were seeded per well and incubated for 48 h. Spontaneous cell sheet assembly of the hEKs was monitored using 10× objective of an inverted microscope (IX73, Olympus, Japan) at 24 and 48 h post-seeding. Cell growth area was measured using the ImageJ software (ImageJ, NIH, Maryland, USA). The hEKs were expanded in the keratinocyte growth medium (MD-0047) provided by iXcells Biotechnolgies, USA.

### F. Immunofluorescence imaging of extracellular matrix proteins

Immunofluorescence imaging was utilized to determine the cellular and extracellular matrix protein expressions in the hDF constructs. The processing of the samples was performed as described previously. Briefly, the scaffolds were first washed with PBS and fixed in 10% neutral buffered formalin for 20 min. The fixed samples were then processed using the standard protocol for paraffinization. The blocks were then cut into 7-*μ*m thick slices and collected on charged slides. The sections were then deparaffinized using xylene followed by rehydration of the section with decreasing gradients of ethanol. Subsequently, the cells were permeabilized with 0.2% Tween^®^20 (Sigma-Aldrich, France) solution in PBS for 30 min (10 min wash 3×) and blocked using 10% NGS (Normal Goat Serum, Invitrogen, UK)/5% BSA/0.3M Glycine (prepared in permeabilizing solution) for 1 h. After blocking, the slides were rinsed in PBS and then incubated at 4 °C overnight with the antibodies to target matrix proteins listed in Table II.

**TABLE II.** List of antibodies used for assessing hDFs differentiation and ECM regeneration in reparative maturation.

Indicators	Primary antibodies (Catalog no.)	Functional roles	Dilutions in 1% BSA solution
Fibroblast markers	Vimentin (ab92547, Abcam, UK)	Mesenchymal filament protein; mediator of fibrosis^61^	1:100
	Alpha-smooth muscle actin (ab7817, Abcam, UK)	Structural filament protein; promotes contraction and scarring^27,62^	1:200
	Fibronectin (ab2413, Abcam, UK)	Provisional matrix protein; promotes fibrosis^63,64^	1:200
Granulation tissue matrix proteins	Collagen III (ab7778, Abcam, UK)	Anti-fibrogenic; Early granulation tissue protein^65^	1:100
	Laminin (ab11575, Abcam, UK)	Nascent basement membrane (BM) protein; BM assembly^66,67^	1:200
	Collagen IV (ab6586, Abcam, UK)	Mature BM protein; BM stability^66^	1:200

After the primary antibody incubation, the slides were rinsed in PBS thrice for 2–3 min each to remove any unbound primary antibodies. Subsequently, the slides were incubated in either Alexa 488-conjugated goat anti-mouse IgG (Cat no. A32723, Invitrogen, UK) and/or Alexa 594-conjugated goat anti-rabbit IgG (Cat no. A11012, Invitrogen, UK) at 1:800 dilution at room temperature for 1 h in the dark. The rinsing step was performed as before and counterstained for nuclei using the mounting medium with DAPI (ab104139, Abcam, UK). The slides were then imaged under a fluorescence microscope (Olympus BX43, Japan) using 20× objective. Samples incubated with only secondary antibodies were used as controls.

#### 1. Image quantification

ImageJ software (ImageJ, NIH, Maryland, USA) was used to semi-quantitatively determine the amount of expressed proteins. For each marker, a constant threshold value was first determined through preliminary imaging of various sections. Using the set threshold value, integrated density (stained area x mean gray value) of the images was measured and then normalized to the number of cells (DAPI counting) to determine a final mean fluorescence density per cell. An average was quantified from 6 random images per nonconsecutive replicate sections (n = 2) per group (n = 3). The averages obtained from replicate sections/group was then used for measuring relative expression between the groups.

### G. Statistical analysis

All results are expressed as mean ± standard deviation. Unpaired, two-tailed t-test was used to calculate the statistical significance between groups, where p< 0.05 was considered to be significant.

## Data Availability

The data that support the findings of this study are available from the corresponding author upon reasonable request.
